# Chagas Disease in the 21st Century: Global Spread, Ecological Shifts, and Research Frontiers

**DOI:** 10.3390/biology14111631

**Published:** 2025-11-20

**Authors:** Marina da Silva Ferreira, Rosa Amelia Maldonado, Priscila Silva Grijó Farani

**Affiliations:** 1 Department of Biological Sciences, Border Biomedical Research Center, The University of Texas at El Paso, El Paso, TX 79968, USA; mdasilvaferre@utep.edu; 2 Department of Pharmaceutical Sciences, School of Pharmacy, The University of Texas at El Paso, El Paso, TX 79902, USA

**Keywords:** Chagas disease, *Trypanosoma cruzi*, global health, vector ecology, oral transmission, congenital transmission, climate change, vector genomics, digital surveillance, neglected tropical diseases

## Abstract

Chagas disease (CD), caused by the parasite *Trypanosoma cruzi*, remains one of the most important neglected tropical diseases. Historically confined to rural areas of Latin America, the disease has now become a global health challenge due to increased migration, urbanization, and ecological changes. This review explores how patterns of transmission and endemicity have evolved, emphasizing the emergence of new geographic hotspots and non-traditional routes of transmission, such as congenital and oral infections. We integrate evidence from ecological studies showing how deforestation, urban sprawl, and climate change are reshaping vector habitats and influencing the spread of triatomine insects. Furthermore, we highlight advances made between 2020 and 2025 in key research areas, including vector genomics and climate-based predictive mapping, as well as digital surveillance strategies that leverage machine learning and citizen science. These innovations provide valuable insights for predicting future risks and improving disease control. By linking global epidemiological trends, ecological drivers, and cutting-edge scientific advances, this review underscores the urgent need for integrated, collaborative strategies to prevent further spread and to protect vulnerable populations worldwide.

## Simple Summary

Chagas disease (CD), caused by the parasite *Trypanosoma cruzi*, has evolved from being a rural disease limited to Latin America into a growing global health challenge. This review describes how changes in human migration, urban expansion, environmental degradation, and weakened health programs have contributed to its spread to non-endemic regions, including North America, Europe, and Asia. It also highlights that transmission routes such as infection through contaminated food and from mother to child during pregnancy are becoming more common, especially in places where the insect that carries the parasite is no longer the main source of infection. Environmental factors such as deforestation and climate change are expanding the habitats of these insects, making control efforts more difficult. Recent scientific advances are providing new tools to fight the disease, including genetic studies of vectors, climate-based mapping to predict risk areas, and rapid, low-cost diagnostic tests. Digital innovations, such as smartphone-based reporting and citizen science projects, are also improving disease surveillance. By integrating ecological, social, and scientific perspectives, this review underscores the need for coordinated global action to prevent further spread and protect vulnerable communities worldwide.

## 1. Introduction

Chagas disease (CD), caused by the protozoan parasite *Trypanosoma cruzi* (*T. cruzi*), has undergone a profound epidemiological transformation in the 21st century [1]. Clinically, CD begins with an acute phase that may present with mild fever, swelling at the infection site, or remain asymptomatic. Over years or decades, about 30–40% of patients develop chronic complications, mainly cardiomyopathy, arrhythmias, and gastrointestinal disorders such as megacolon and megaesophagus [2]. Current treatment relies mainly on benznidazole and nifurtimox, which are most effective in the acute phase but often limited by toxicity and reduced efficacy in chronic infections [1]. Once considered a strictly Latin American disease, CD has expanded far beyond its traditional borders due to global migration, urbanization, and ecological disruption [3]. This review aims to provide an updated perspective on the current landscape of CD, integrating recent evidence and highlighting critical developments from 2020 to 2025.

In the Section 2, Global Spread, we explore how patterns of transmission and endemicity have shifted, detailing how human migration, socio-economic changes, and the breakdown of control programs have facilitated the emergence of CD in new geographic regions. To provide a broader view of transmission dynamics, this section also introduces non-vectorial routes, particularly oral and congenital infection, that have gained prominence in both endemic and non-endemic settings. These silent transmission pathways remain underreported due to the predominance of vector-centered surveillance and highlight the need for more integrated epidemiological frameworks that encompass the full transmission cycle and current treatment challenges. The Section 3, Ecological Shifts, delves into the environmental and biological drivers behind these changing patterns. Here, we discuss how climate change, deforestation, urban sprawl, and vector adaptation are reshaping the epidemiology of *T. cruzi* transmission. Particular attention is given to insecticide resistance, operational challenges in vector control, and the emergence of new ecological niches that blur the boundaries between sylvatic, peridomestic, and domestic transmission cycles. Finally, in the Section 4, Research Frontiers, we compile cutting-edge studies published between 2020 and 2025 that are advancing our understanding of CD. This includes innovations in vector genomics and surveillance, climate-based predictive mapping, and diagnostic tools, as well as digital surveillance strategies that integrate machine learning and citizen science. Together, these research frontiers showcase the transformative potential of new technologies and approaches for controlling CD and mitigating its global impact.

By linking global epidemiological shifts, ecological drivers, and scientific advancements, this review provides a comprehensive framework for understanding how CD is evolving and how public health responses must adapt to meet the challenges of the coming decade.

## 2. Global Spread

Chagas disease is now recognized as a major global public health problem. It affects an estimated 6–7 million people worldwide, places about 75 million individuals at risk of infection, and causes approximately 10,000 deaths annually [1,2]. Traditionally associated with rural areas of Latin America and commonly known as American trypanosomiasis, the disease has expanded into urban areas and non-endemic countries, including the United States, Spain, Italy, and Japan [4]. The global spread is no longer primarily sustained by classical domiciliary vector-borne transmission, which has been reduced in many endemic regions, but rather by non-vectorial pathways, particularly oral and congenital transmission in both endemic and non-endemic settings [5,6]. Cases have been increasingly detected in non-endemic regions such as Europe and North America, where chronic cardiac manifestations have been documented among migrants and travelers [7,8]. In these contexts, cases are often diagnosed only during the chronic phase as CD cardiomyopathy [9,10]. The expansion has been driven by human migration and globalization, while urbanization, poor housing conditions, and poverty remain significant contributors, particularly in peri-urban slums with inadequate vector control [11,12,13].

### 2.1. Latin America: The Historic Epicenter

First identified by Carlos Chagas in Brazil over a century ago [14], CD remains deeply entrenched in Latin America, where it continues to exact a disproportionate toll on vulnerable populations [15,16]. The Southern Cone Initiative (1991) was a pivotal regional effort that significantly reduced domiciliary vector transmission through coordinated vector control, systematic indoor residual spraying, and blood donor screening [17,18]. Additional measures have included improved diagnostic screening of blood donors and pregnant women, antiparasitic treatment with benznidazole or nifurtimox, and ongoing research into novel drugs and vaccine candidates [1,2]. Such coordinated actions, particularly effective in Argentina, Brazil, Uruguay, and Chile, were initially celebrated as major public health successes [18].

Yet regional disparities persist. Bolivia continues to have the highest national prevalence (>6%, with pockets of even higher burden), especially in Cochabamba and Santa Cruz, where rural poverty, limited healthcare, and poor housing sustain transmission [19,20]. In Brazil, the suppression of classical vector-borne transmission mainly reflects the successful elimination of the domestic vector *Triatoma infestans* from the Southern and Central regions. However, in the Amazonia basin, oral transmission has emerged as a growing concern, with recurrent outbreaks linked to food contamination by feces of sylvatic triatomine species, particularly during the processing of açaí juice and sugarcane extract [21,22,23,24]. Meanwhile, Argentina and Paraguay have achieved substantial reductions through spraying campaigns, though peri-urban hotspots characterized by informal housing, population mobility, and environmental changes such as deforestation, agricultural expansion, and urban sprawl, as well as patchy surveillance remain difficult to eliminate [25].

The persistence and resurgence of CD in endemic regions is driven by a complex interplay of environmental, political, and infrastructural dynamics [25,26]. Wild triatomines increasingly adapt to human-modified landscapes [9,11], and deforestation, fragmentation, and climate change accelerate their colonization of peridomestic and domestic settings [9,21]. In addition, hybridization events among *Rhodniini* species, particularly between *Rhodnius prolixus* and *Rhodnius robustus*, have been reported, suggesting gene flow that may enhance vectorial capacity and ecological adaptability under changing environmental conditions [27]. Recent evidence suggests that hybridization among *Rhodniini* species may enhance vectorial capacity, raising new concerns in the context of anthropogenic change [28,29]. These risks are compounded by the erosion of vector control programs since 2010, with weakened surveillance, reduced insecticide spraying, and declining community engagement [30,31]. At the same time, health systems, especially in remote and indigenous areas, lack trained personnel, diagnostic tools, and access to trypanocidal drugs [15,26,32]. Underfunding and political marginalization continue to hinder sustained progress [33,34].

### 2.2. Emergence in Non-Endemic Regions: A Global Health Challenge

CD has undergone a marked epidemiological transformation, spreading to North America, Europe, Asia, Oceania, and Australia [34]. With increasing migration from Latin America to multiple continents, *T. cruzi* has silently crossed borders, exposing health systems to a neglected but increasingly global parasitic infection [10,35,36,37,38].

In the United States, over 300,000 people are estimated to be chronically infected, mostly due to migration from endemic regions [37,39]. However, diagnosis remains rare due to limited physician awareness, underreporting, and the absence of routine screening [37]. Recent estimates suggest that fewer than 1% of infected individuals in the U.S. have been diagnosed or treated, underscoring the persistent invisibility of the disease even in high-resource settings [39]. A systematic review by Bern et al. (2019) revealed gaps in clinical recognition and access to treatment, even in cardiology clinics where patients may present with arrhythmias or heart failure, typical of CD cardiomyopathy [7]. Beyond congenital and transfusion-related pathways, sylvatic vector-borne transmission is increasingly documented in the southern United States [40]. A comprehensive surveillance study by Rodriguez et al. (2021) documented *T. cruzi* infection in triatomine vectors, feral dogs, cats, and wild mammals in and around El Paso County, Texas, and New Mexico, highlighting the presence of established enzootic cycles [9]. The study identified *Triatoma gerstaeckeri* and *Triatoma rubida* as the primary vectors maintaining parasite circulation in peri-urban and ecotonal habitats where wildlife, domestic animals, and humans overlap [9]. While confirmed autochthonous human infections remain rare, these ecological dynamics pose a significant but underrecognized public health risk. Congenital transmission is another overlooked route [41]. Edwards et al. (2019) emphasized that early detection and treatment in neonates born to infected mothers is highly effective, and they called for routine screening of pregnant women from endemic countries [42]. Nevertheless, implementation remains limited, and CD is still excluded from U.S. national prenatal screening programs, despite being classified as a “neglected parasitic infection” by the Centers for Disease Control and Prevention (CDC) [37].

In Europe, Spain, Italy, and Switzerland carry the largest burden of imported cases, yet screening protocols for blood donation, pregnancy, and transplantation remain fragmented [8,35]. The absence of an EU-wide surveillance system has hindered coordinated control efforts [35]. Although Spain has implemented regional perinatal screening programs in Catalonia and Valencia, national-level policies remain absent, and most other European countries rely on ad hoc or voluntary protocols [36]. In Spain, Navarro et al. (2022) and Gómez i Prat et al. (2019) have called for urgent public health interventions, pointing to multiple cases of undiagnosed CD cardiomyopathy that led to avoidable complications [32,38].

In Japan, imported cases have been reported primarily among Latin American immigrants and returning nationals [43,44]. While no autochthonous transmission has been observed, Xu et al. (2024) identified *T. dionisii* in bats in Japan and China, raising concern about environmental suitability for future spillover events [45]. Japan’s role in drug development and global health cooperation continues to grow, but clinical awareness remains low domestically [46]. Across Asia and Africa, Picanço et al. (2024) reported the emergence of triatomine vectors in new ecological niches, driven by climate change and urbanization [47]. Climate-based ecological niche modeling further suggests that southern Europe, Southeast Asia, and parts of the southern United States may become increasingly suitable for triatomine colonization under projected warming scenarios [28,29]. Though human transmission has not yet been confirmed, these shifts suggest the ecological groundwork for possible future expansion.

In Australia, CD is seen primarily among migrants and travelers, yet general practitioners often fail to recognize it, and screening for blood donors is inconsistent [48]. Despite the lack of native triatomines, gaps in migrant health protocols mean many infections go undetected or misdiagnosed [49,50]. Bed bugs have been experimentally shown to transmit *T. cruzi* to mice [51]. Reports of bed bug infestations within the country raise concerns about the potential for these insects to contribute to the autochthonous transmission of the parasite [52,53].

Encouragingly, non-endemic countries are increasingly contributing to therapeutic innovation and global policy responses. The United States, for example, has advanced preclinical studies on pyronaridine, a repurposed antimalarial that reduced parasite burden in mouse models of chronic CD, offering new hope for patients for whom benznidazole and nifurtimox are inadequate or inaccessible [54]. In parallel, Europe and Japan are leading multicenter trials of new trypanocidal regimens and drug combinations, reflecting a growing global research agenda that aligns with the WHO 2021–2030 roadmap goals of expanding treatment options and overcoming the current limitations of benznidazole and nifurtimox [55,56]. However, innovation alone cannot overcome the structural drivers of CD, which remain deeply rooted in poverty, migration, ecological disruption, and global health inequity [33,34]. Despite scientific progress, the disease continues to disproportionately affect marginalized populations who remain underserved by health systems [19].

**Figure 1:**
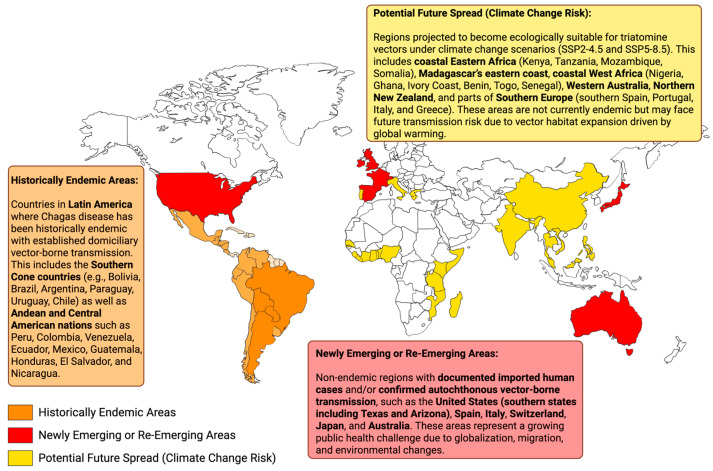
Global distribution of CD, categorized by historically endemic areas (orange), newly emerging or re-emerging areas (red), and regions projected to become ecologically suitable for triatomine vectors under climate change scenarios (yellow) [1,3,69].

To confront this expanding global threat, non-endemic host countries must complement innovation with sustained public health action. Spain, Italy, and Switzerland have piloted or implemented routine screening of pregnant women and blood donors, though policies remain fragmented and lack regional harmonization [8,57,58,59]. Spain and the United States have been leaders in integrating CD into clinical protocols, particularly in cardiology and migrant health services [7,38]. At the European Union level, initiatives, especially in Spain, France, and the United Kingdom, have expanded ecological and entomological surveillance in areas predicted to become suitable for triatomine vectors under climate change scenarios [9,47]. In parallel, regional networks in Europe and North America have strengthened cross-border collaboration in diagnostics, treatment access, and health workforce training [32,35]. Finally, both European countries and the United States have provided research funding and public health investment to complement national programs in endemic Latin American countries [60,61]. Unless addressed holistically, CD will continue to elude detection, deepen global health inequities, and impose an unnecessary burden on underserved populations worldwide [34]. The global distribution of CD is illustrated in Figure 1, showing historically endemic areas, newly emerging regions, and areas projected to become suitable for triatomine vectors under climate change.

### 2.3. Oral Transmission: A Growing Driver of Global Outbreaks

Oral transmission has emerged as the dominant acute pathway in recent decades, especially in Amazonia and northern South America [5,62,63]. Unlike classical vector-borne transmission via triatomine feces deposited on skin or mucosa, oral transmission occurs through ingestion of parasite-contaminated food or beverages [1]. In this context, sylvatic triatomine species play an indirect but central role, as their infected feces can contaminate food products during collection, processing, or storage. Foods such as açaí pulp, sugarcane juice, bacaba, guava, and cassava are particularly implicated because they are often processed manually, stored at ambient temperatures, and prepared in open environments where contact with infected triatomines or their feces is possible. These conditions provide ideal opportunities for contamination, especially in small-scale or artisanal production systems without strict sanitary controls [25]. In Brazil, it is now estimated that over 70% of newly reported acute cases are linked to oral exposure, highlighting its central role in contemporary epidemiology [24]. This pathway is now responsible for most acute CD cases in the Amazonia region and has been linked to severe outbreaks in Brazil, Venezuela, Colombia, and French Guiana [5,21,22,24,63].

Numerous large-scale outbreaks have reshaped the epidemiological landscape of CD [64]. In 2007, one of the most severe outbreaks occurred in Barcarena, Pará State, Brazil, where over 100 people were infected after consuming contaminated açaí juice [65]. Other major Brazilian outbreaks followed in Amapá (2010), Amazonia (2014), and Tocantins (2016), all involving food products such as açaí, bacaba juice, or sugarcane [24]. In Venezuela, outbreaks in the Andean and central regions affected schoolchildren and families who consumed contaminated guava juice and cassava [66]. A notable event in Chacao, Caracas (2007) caused over 100 infections and multiple deaths, primarily in children [66]. In Colombia, several outbreaks of orally transmitted CD have been reported, particularly in the departments of Casanare and Boyacá, where the consumption of contaminated food and fruit juices has been implicated [67,68]. These outbreaks have been marked by acute clinical presentations, including myocarditis, and in some instances have shown case-fatality rates exceeding 40%, underscoring the aggressive nature of oral transmission and highlighting the need for strengthened food safety and surveillance systems to prevent future episodes [63].

The oral transmission outbreaks, particularly those reported in Brazil, Venezuela, and Colombia, share consistent clinical features that illustrate the severity of this transmission route. They are characterized by rapid onset of symptoms, high hospitalization rates, and elevated mortality [66]. The clinical pattern is largely due to the distinct immunological response elicited by oral transmission compared to vectorial infection [65]. When *T. cruzi* is ingested, it bypasses the natural skin barrier and directly encounters the gastrointestinal mucosa [70]. This leads to higher parasite loads (parasitemia) and a broader mucosal immune activation, which triggers a more intense and systemic pro-inflammatory response [70,71]. Studies in both humans and animal models show that orally acquired infections lead to increased levels of TNF-α, IFN-γ, IL-6, and nitric oxide, which contribute to acute tissue inflammation, particularly in the heart, liver, and gastrointestinal tract [24,72]. This contrasts with vectorial infections, where the immune response is typically more localized and less overwhelming due to a smaller parasite inoculum and slower dissemination [73,74]. As a result, oral cases often present more extensive myocarditis, pericardial effusion, and occasionally meningoencephalitis, particularly in younger patients or those with delayed treatment [15]. Histopathological studies of autopsy samples from fatal oral cases reveal dense myocardial infiltrates, extensive necrosis, and parasite nests within cardiac fibers, and evidence of the overwhelming immune-pathogen confrontation [65]. Conversely, vectorial infections tend to elicit a slower and more contained immune response, resulting in milder symptoms and lower early mortality, though both routes can lead to chronic complications if untreated [75]. From a clinical perspective, orally transmitted CD is typically characterized by high, sustained fever, generalized edema, hepatosplenomegaly, severe myocarditis or pericarditis, elevated liver enzymes and lymphocytosis, and rapid clinical deterioration in some cases [76].

The combination of high parasite burden, systemic inflammation, and limited access to diagnostics in rural outbreak pulp, enforcement is inconsistent, especially in remote or informal markets [77]. The settings contributes to the higher lethality of oral transmission, with reported mortality rates ranging between 10% and 35% depending on region and age group [66]. Despite the severity and frequency of these events, surveillance systems remain underdeveloped [9,35]. Many countries lack structured protocols for identifying foodborne outbreaks, and oral transmission is not consistently integrated into CD control strategies [77]. Environmental drivers such as deforestation, expansion of agricultural frontiers, and poor sanitary control in artisanal food chains, especially in the açaí production system, further increase the risk of oral outbreaks [21,22,24]. While Brazil has implemented heat treatment standards for açaí World Health Organization (WHO) Neglected Tropical Disease (NTD) Roadmap 2021–2030 now recognizes oral transmission as a critical challenge, calling for improved surveillance, food safety policies, and integration into national control strategies in both endemic and non-endemic regions [56].

In summary, oral transmission represents both a clinical and public health challenge that differs significantly from vectorial pathways [65]. It provokes a more severe and inflammatory immune response, presents greater diagnostic and therapeutic urgency, and requires food safety interventions and robust surveillance to prevent future outbreaks [5].

### 2.4. Congenital Transmission: A Silent Global Threat

Congenital transmission of *T. cruzi*, from infected mother to fetus, has emerged as a key public health concern in both endemic and non-endemic settings [31,41,42]. The transmission route circumvents vector contact entirely, making it particularly relevant in non-endemic countries where triatomine bugs are absent but Latin American migration is substantial [8]. Although, despite its preventability and the availability of effective treatments, congenital CD remains underdiagnosed and largely invisible in routine maternal-child health systems [6], global estimates suggest that 8000–15,000 new congenital cases occur annually, making it the second most important transmission route worldwide after vectorial exposure [78].

Epidemiological studies estimate that 1% to 10% of chronically infected mothers transmit the parasite to their infants, with variation depending on maternal parasitemia, immune status, gestational stage, and parasite genotype [79,80]. A prospective multi-country study conducted in Argentina, Mexico, and Honduras confirmed that congenital transmission accounts for a growing proportion of CD incidence in the absence of vectorial transmission [81]. In high-risk populations, such as migrant women from Bolivia and Brazil, transmission rates can reach 5–7%, particularly in areas without antenatal screening protocols [82]. Other endemic countries, including Paraguay and Colombia, also report substantial congenital transmission, reinforcing its importance as a regional health challenge [83].

Infants born with congenital CD are often asymptomatic at birth, which complicates detection [78]. When symptoms are present, they may include low birth weight, hepatosplenomegaly, anemia, thrombocytopenia, and less commonly, myocarditis or meningoencephalitis [41,42]. In the absence of early treatment, many of these children develop chronic complications in adolescence or adulthood, including dilated cardiomyopathy, conduction abnormalities, and digestive mega-syndromes [41]. When symptoms do appear, they may include hepatosplenomegaly, anemia, thrombocytopenia, low birth weight, myocarditis, and in rare cases, meningoencephalitis [6]. Delayed treatment allows *T. cruzi* to persist and disseminate silently, predisposing the child to chronic cardiac or gastrointestinal complications decades later [42,83]. Furthermore, congenital CD can alter immune development, resulting in persistent immune activation even after treatment [41]. The “immunological imprinting” may influence vaccine responsiveness and susceptibility to other infections throughout life [84]. In some cases, chronic antigen stimulation by persisting parasites can result in T-cell exhaustion, reduced proliferative capacity, and diminished IFN-γ production, thereby impairing the ability to clear the parasite in the long term [26,32].

Recent research highlights key immunological implications for congenitally infected neonates. Affected newborns typically mount an early Th1-type immune response, characterized by elevated interferon-gamma (IFN-γ) and tumor necrosis factor-alpha (TNF-α), like acute infections in adults [31]. However, immature neonatal immunity and reduced regulatory cytokines like IL-10 can make this inflammatory response poorly controlled, leading to tissue damage, especially in the myocardium and liver [83]. Some infants may also show elevated markers of endothelial activation and systemic inflammation, which correlate with disease severity and outcomes [83].

The international health authorities have issued renewed calls for systematic maternal screening [6]. The World Health Organization (WHO) and Pan American Health Organization (PAHO) recently urged countries to incorporate routine antenatal testing for women from endemic areas into national maternal health programs [82,85]. The WHO NTD Roadmap 2021–2030 explicitly identifies the elimination of congenital CD transmission as a core target, reflecting its preventable nature and the effectiveness of maternal screening combined with early treatment [55,56]. In Spain, where Latin American migrants form a substantial portion of the population, Navarro et al. (2022) highlighted the cost-effectiveness and public health benefit of implementing such screening strategies, though they remain limited to certain autonomous regions and lack national coordination [32]. Pilot programs in Brazil and Italy have also demonstrated the feasibility of integrating CD testing into existing antenatal care structures [6,41].

In the United States, Edwards et al. (2019) and Bern et al. (2019) advocated for universal prenatal risk assessment and diagnostic follow-up of infants born to seropositive mothers [7,42]. Also, awareness among obstetricians and pediatricians remains low, and congenital CD is not integrated into most prenatal or pediatric care algorithms [37,83]. Barriers are further compounded by migrant women’s limited access to healthcare due to legal, linguistic, and socioeconomic vulnerabilities, leading to underdiagnosis and missed opportunities for early intervention [8,86].

Diagnosis of congenital cases is further complicated by limited access to reliable laboratory testing. Although PCR-based diagnostics have shown high sensitivity, particularly within the first six months of life, their availability is constrained in many healthcare settings [6]. In addition, Requena-Méndez et al. (2015) identified major gaps in laboratory capacity and institutional support across Europe, calling for enhanced diagnostic infrastructure [86]. Recent efforts to validate rapid diagnostic tests (RDTs) for use in antenatal screening may offer a scalable alternative, but further validation in field conditions is required [6].

Despite decades of evidence, most countries have not implemented national strategies for preventing congenital CD [10,26,79]. Surveillance is fragmented, data is sparse, and political prioritization remains limited [30]. Still the path forward is clear: early detection and treatment are nearly 100% effective in infants, and maternal screening can significantly reduce future disease burden [78,83]. Integration of CD testing into routine antenatal care, particularly in migrant health frameworks, should be seen as a public health imperative [6]. An overview of the main transmission routes, their mechanisms, key epidemiological data, and public health implications is provided in Table 1.

## 3. Ecological Shifts

### 3.1. Environmental and Ecological Factors in Vector Spread

The ongoing transmission of *T. cruzi* remains deeply tied to the ecology and behavior of triatomine vectors [11,31]. Environmental transformation, particularly deforestation, land conversion for agriculture, and peri-urban sprawl, has dramatically reshaped the epidemiological landscape in endemic Latin American regions [87]. One of the most significant consequences is the adaptation of sylvatic triatomines to human-modified environments [88]. As highlighted by Rengifo-Correa et al. (2021), fragmented forests have led sylvatic vectors to increasingly colonize peridomestic and domestic ecotopes, defined, respectively, as natural habitats of wild animals and vectors, adjacent human structures such as animal shelters or storage areas, and human dwellings themselves, thereby intensifying human–vector contact [89]. In areas like the Amazonia basin and the dry Gran Chaco, triatomines are now frequently found in thatched roofs, wall crevices, and nearby animal shelters, zones where structural vulnerability is high and vector control is limited [12,90].

Historically, *T. cruzi* transmission was linked to rural, impoverished households, but this dynamic has evolved. Species such as *Rhodnius neglectus* and *Triatoma brasiliensis* have exhibited urban colonization patterns, now being identified in city parks, peri-urban structures, and even high-density neighborhoods in Brazil and Bolivia [27,91]. Recent surveys also report peri-urban dogs and cats acting as blood meal sources for triatomines, reinforcing their role as urban reservoirs that sustain parasite circulation [9,87,92]. These species, once considered strictly sylvatic, now bridge the divide between wild and urban habitats, challenging traditional assumptions about CD being a rural affliction [21]. The ecological shift has also occurred in non-endemic regions, such as the southern United States [93]. Rodriguez et al. (2021) demonstrated the presence of *T. cruzi*-infected triatomines in El Paso County, Texas, and New Mexico, as well as infection in feral dogs, cats, and wild mammals [9]. These findings confirm established sylvatic cycles and suggest that human encroachment into ecotones increases the likelihood of zoonotic spillover, a growing concern amid urban expansion [90]. Furthermore, ecological niche models project that rising temperatures may expand the potential range of *Triatoma gerstaeckeri* and *Triatoma sanguisuga* further north, raising the likelihood of future autochthonous transmission in areas previously considered low risk [28,94].

Another important example is Australia, where although no native triatomine vectors are present, imported cases of CD have steadily increased among Latin American migrants and travelers. Some studies report growing concern over potential vector introduction through global trade (e.g., contaminated shipping containers or wooden products), which could theoretically introduce triatomine species to Australian environments [48,95]. While sustained transmission has not been observed, the lack of mandatory screening for at-risk individuals and low clinical awareness make early detection and containment challenging [1]. This case illustrates how globalization and ecological interconnectivity can create transmission risks even in countries with historically no endemicity [8,10,50]. Furthermore, climate change is suspected to alter triatomine distributions. Rising temperatures and changing rainfall patterns may facilitate the northward migration of vectors or allow existing populations to persist longer during seasonal cycles, effectively expanding the transmission window [47]. Coupled with this, emerging evidence of insecticide resistance in *T. infestans* populations of the Gran Chaco threatens the sustainability of control programs [90]. Additionally, experimental studies of *Rhodniini* hybrids indicate that anthropogenic pressures may favor the emergence of lineages with enhanced vectorial competence [28].

**Figure 2:**
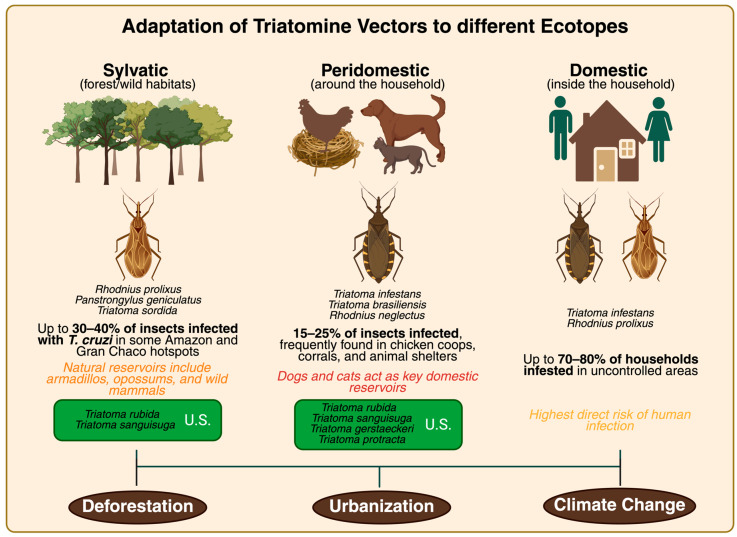
Ecological niches of triatomine vectors and their role in Chagas disease transmission. Sylvatic species such as *R. prolixus*, *P. geniculatus*, and *T. sordida* show infection rates of up to 30–40% in some Amazonia and Gran Chaco hotspots, with *T. rubida* and *T. sanguisuga* specifically documented in Texas, indicating active natural transmission cycles in wildlife habitats. In peridomestic settings, species including *T. infestans*, *T. brasiliensis*, *R. neglectus*, *T. gerstaeckeri*, *T. protracta*, and *T. sanguisuga* are frequently associated with human dwellings, chicken coops, corrals, and domestic animals such as dogs and cats as reservoirs. Studies in El Paso, Texas, and New Mexico have highlighted *T. gerstaeckeri*, *T. rubida*, and *T. protracta* as key peridomestic vectors, while data from central and northern Texas also implicate *T. sanguisuga* as an important species in human and animal contact zones. Domestic colonization by *T. infestans* and *R. prolixus* may reach 70–80% of households in the absence of control, representing the highest risk of direct human transmission. Processes such as urbanization, deforestation, and climate change further drive these ecological shifts, blurring the boundaries between sylvatic, peridomestic, and domestic cycles.

In summary, ecological and climate pressures have resulted in a blurring of boundaries between sylvatic, peridomestic, and domestic transmission zones, and control strategies must adapt accordingly [12,13]. The distribution of triatomine infection rates across different ecological niches is summarized in Figure 2.

### 3.2. Insecticide Resistance and Operational Challenges

While the Southern Cone Initiative and subsequent vector control programs have historically relied on pyrethroid-based indoor residual spraying (IRS), recent years have seen increasing evidence of vector resistance, threatening the backbone of CD suppression strategies [12,13,18,35].

Triatomine resistance to pyrethroids is now widely documented in Argentina, Bolivia, Paraguay, and parts of Brazil, with *Triatoma infestans* being the most affected species [96,97,98]. Resistance arises through multiple mechanisms, including metabolic detoxification via increased expression of cytochrome P450 enzymes, target-site mutations in voltage-gated sodium channels, and behavioral adaptations, such as avoidance of sprayed surfaces or altered nocturnal feeding patterns [99]. Recent studies have shown that some vector populations display up to 50% resistance prevalence, with resistance gradients strongly linked to agrochemical use, habitat fragmentation, and inconsistent spraying campaigns [13,100]. Roca-Acevedo et al. (2025) conducted a meta-analysis demonstrating regional variability in resistance emergence, suggesting that resistance is not evenly distributed but rather concentrated in areas where selective pressure is high and surveillance weak [100].

Moreover, reports from the Gran Chaco highlight not only pyrethroid resistance but also emerging reduced susceptibility to organophosphates and carbamates, raising concerns about cross-resistance and the sustainability of current chemical options [101]. New field trials in Bolivia and northern Argentina (2022–2023) indicate that IRS efficacy has declined significantly in resistant foci, prompting WHO and PAHO to recommend exploring rotation schemes, insecticide mixtures, and alternative formulations such as long-lasting insecticidal paints and impregnated nets [12,55,56].

Adding to the challenge is the difficulty of detecting low-density vector infestations in peri-urban and semi-urban environments [21,93]. These “silent infestations” often occur in hidden microhabitats such as beneath roofing materials, behind walls, mattresses, and in utility spaces. Standard surveillance tools, such as timed-manual searches and community reporting, often fail to identify these vectors [102]. In many cases, by the time an infestation is identified, transmission may have already occurred, or re-colonization after spraying may have been silently underway [102,103]. While chemical control remains a central strategy, alternative and complementary measures that reduce selective pressure for insecticide resistance are increasingly important. These include biological control using entomopathogenic fungi, integrated environmental management to reduce vector habitats [104]. Furthermore, household construction trends in marginal neighborhoods, such as the use of porous materials, lack of plastering, and informal expansion, create ideal habitats for triatomine colonization [93]. These vulnerabilities are amplified by rapid unplanned urbanization, climate variability, and the weakening of community-based vector surveillance networks after 2010, which together undermine sustained suppression of domestic and peri-domestic transmission [87]. Thus, vector resurgence is not only a biological issue but also a social and structural one, deeply entangled with housing inequality, and extensive urbanization haven as consequence the triatomines loss of the ecological niche [12,93].

### 3.3. Innovations and Strategic Needs

Given the intersection of ecological change, biological resistance, and operational fragility, traditional vector control paradigms must evolve [12]. Current strategies require the integration of novel technologies, predictive tools, and participatory approaches to remain effective [55,56]. These innovative strategies should be framed within a One Health perspective, which recognizes the interconnection between human, animal, and environmental health as a foundation for effective, sustainable control of vector-borne diseases [19].

Firstly, new insecticides alternatives must be urgently developed and validated. Promising candidates include neonicotinoids, phenylpyrazoles, and growth regulators, although their field deployment remains limited [13]. Rotational spraying, using insecticides with different modes of action, may help delay resistance onset, but requires well-coordinated surveillance systems [100]. Recent field trials in Bolivia and Paraguay have also tested long-lasting insecticidal paints and impregnated bed nets, which show potential for sustained protection in peri-domestic structures where conventional IRS efficacy is declining [103].

Secondly, enhanced detection tools are essential. Molecular xenomonitoring of triatomine DNA or *T. cruzi* parasite loads, which involves detecting parasite DNA within insect vectors to monitor transmission risk, offers high-sensitivity surveillance that can detect infestations before they are clinically apparent [105]. Spatial modeling, remote sensing, and machine learning tools can be applied to predict vector hotspots based on environmental and demographic variables [106]. Additionally, digital surveillance has proven feasible even where formal systems are weak. In Venezuela, the #TraeTuChipo (“Bring Your Kissing Bug”) citizen-science campaign (launched in January 2020) implemented real-time, app/social-media/SMS-based entomological reporting and georeferencing, documenting 79 triatomines across 18 states, predominantly *Panstrongylus geniculatus*, with qPCR-confirmed *T. cruzi* TcI in fecal samples and frequent human blood meals, despite the absence of a functional national surveillance program [107]. Also, smartphone-based entomological reporting systems have been piloted in Brazil, demonstrating that community-generated data can improve response times and expand the geographic reach of surveillance [108,109,110]. Despite their potential, these innovations face limitations including cost barriers, the need for trained personnel, and limited applicability in low-resource settings, which may constrain large-scale implementation [110].

Thirdly, community-based strategies must be revitalized [12,13]. While many national programs have emphasized spraying and diagnostics, community education and empowerment, including participatory entomological monitoring and early warning systems, have been neglected in recent years [1,2]. These are particularly important in peri-urban areas, where traditional public health outreach has often failed to keep pace with rapid urban growth [21,87,88]. Novel participatory models, such as eco-health approaches that integrate vector control with housing improvement and environmental management, have proven effective in reducing reinfestation in rural Guatemala and Honduras, and could be scaled up in other endemic regions [111]. In parallel, advances in molecular surveillance, such as the 2b-RAD approach proposed by Hernández-Castro et al. (2017), provide cost-effective tools to strengthen population monitoring of *Triatoma* vectors, complementing community-based and One Health initiatives [112]. Community-based eco-health and surveillance models embody the practical application of One Health, fostering collaboration among public health, veterinary, and environmental sectors to address the social and ecological determinants of transmission.

Ultimately, cross-sectoral coordination with urban planners, housing programs, and environmental management agencies will be crucial to developing sustainable, integrated vector management strategies [12]. Vector control can no longer be confined to spraying campaigns; it must address the underlying structural, social, and ecological determinants of disease transmission [13]. Integration with the WHO 2030 roadmap priorities, particularly housing improvement, climate adaptation, and universal access to diagnostics, will be essential to align operational innovations with long-term elimination goals [56].

## 4. Research Frontiers

### 4.1. Advances in Vector Surveillance and Ecology

Genomic, Transcriptomic, and Proteomic Profiling of Triatomine Vectors

Recent omics studies have greatly expanded our understanding of triatomine vectors’ biology and their interactions with *T. cruzi*. Emerging surveillance methods such as environmental DNA (eDNA) detection are providing new, non-invasive approaches to identify triatomine presence in domestic and peridomestic habitats [113]. Genomic sequencing has progressed with the first high-quality assemblies for key species, including the whole genome of *Triatoma sanguisuga* (a major North American vector) [114] was completed in 2025, revealing a 1.16 Gb genome with ~17,799 predicted genes [115]. This assembly is the most complete Triatominae genome to date (99.1% BUSCO completeness) and is rich in repetitive DNA (~61% of the genome) [116]. The new genomic resource provides a foundation to explore traits like blood-feeding, host-seeking, and parasite competence, offering potential targets for vector control interventions.

Moreover, transcriptomic analyses using next-generation sequencing (NGS) have profiled gene expression across vector life stages, tissues, and stress responses, providing insights into metabolic regulation, immunity, and adaptation mechanisms in *Triatoma* species. For example, an RNA-seq study of *Triatoma rubrofasciata* across all developmental stages identified stage-specific gene expression patterns [116]. Genes related to immunity and metabolism were differentially expressed during development, and intriguingly, venom-like salivary proteins (e.g., histidine phosphatase and serine carboxypeptidase homologs) were highly upregulated in late nymphal instars. These venom-associated proteins likely facilitate blood-feeding by modulating the host at the bite site. In adult triatomines, a cytochrome P450 (CYP425A1) showed the highest expression, suggesting enhanced detoxification capacity in the adult stage [116]. Such P450 upregulation is thought to reflect metabolic adaptation for processing toxins or insecticides, underscoring a link between gene expression and detoxification mechanisms in vectors. Similarly, transcriptomic profiling of *T. infestans* after sub-lethal deltamethrin exposure revealed a complex stress response: numerous genes in ABC transporter families, heat shock proteins, odorant-binding proteins, and cuticle formation were differentially expressed. This global response illustrates how vectors mobilize multiple detoxification pathways and protective measures under chemical stress [117], providing insight into potential insecticide resistance mechanisms.

Proteomic and sialomic studies with proteomics and transcriptomics integration of salivary glands have illuminated the protein arsenal that triatomines use to obtain blood and evade host defenses. Triatomine saliva is a cocktail of anti-hemostatic and immunomodulatory factors, and high-throughput analyses confirm its composition. In *Triatoma sordida*, the first comprehensive sialome profiling identified >26,000 salivary transcripts and 132 secreted proteins [118]. Lipocalin-family proteins (e.g., triatin, triafestin) emerged as the dominant class in saliva, a finding consistently reported across species, serving to prevent blood clotting or inflammation. Indeed, in *T. rubrofasciata* salivary transcripts, ~85% of total expression corresponded to secreted proteins, with lipocalins accounting for ~89% of that secretory output [119]. These abundant lipocalins include anti-clotting factors like pallidipin and anti-inflammatory agents like triafestin that help the bug feed efficiently [119]. Other salivary proteins identified in high abundance include apyrases and protease inhibitors (Kazal-type serine protease inhibitors, antigen-5/CAP family proteins), which further contribute to anticoagulation and immune evasion at the bite site [119].

Other omics studies are linking vector immune factors to parasite survival, shedding light on vector competence. A comparative proteomic analysis of hemolymph and salivary glands in *R. prolixus* versus *R. colombiensis* (a related sylvatic species) revealed species-specific differences in immune protein abundance [120]. *R. prolixus*, a highly competent vector, had significantly higher levels of trypanolytic molecules (including lysozyme, prolixin, nitrophorins, and serpins) in its hemolymph compared to *R. colombiensis* [120]. Likewise, *R. prolixus* saliva was richer in nitrophorins, lipocalins, and triabins (heme-binding and immune-modulating proteins). These differences correlate with the ability to kill or restrict certain *T. cruzi* strains: *R. prolixus* hemolymph can lyse parasites belonging to TcII (Y strain), whereas *R. colombiensis* cannot. The findings suggest that *R. prolixus*’s higher constitutive immune arsenal imposes oxidative and nitrosative stress on parasites, to which some strains (*T. cruzi* TcI, e.g., Dm28c strain) are evasion-adapted. This mechanism of parasite immune evasion in vectors (by resisting the vector’s antimicrobial peptides and oxidative attack) may partly explain the differing geographic distributions of *T. cruzi* strains [120,121]. Overall, such integrative omics studies highlight candidate genes and proteins, from detoxifying P450s to salivary lipocalins and gut immune factors, that are critical to vector competence, feeding success, and insecticide survival in CD vectors. Key recent omics studies on triatomine vectors are summarized in Table 2, highlighting advances in genomics, transcriptomics, and proteomics.

Climate-Based Predictive Mapping of Triatomine Vector Distribution

Innovations in geospatial modeling are now being applied to predict where triatomine vectors might thrive under current and future climates. Machine learning (ML) algorithms, ecological niche modeling (ENM), remote sensing, and GIS-based spatial analyses are revolutionizing risk mapping for CD. Recent studies leverage large species occurrence datasets alongside climatic and environmental variables to forecast changes in vector distribution and to pinpoint high-risk areas for vector invasion. New technologies for vector monitoring include remote-sensing tools for habitat mapping, artificial intelligence-based image recognition for insect identification, and smart traps equipped with sensors that capture real-time vector activity data [122,123,124].

A landmark study by Brasil et al. (2025) [69] used an ensemble niche modeling approach (including ML algorithms like MaxEnt and random forest) to project the distributions of 55 triatomine species across Latin America under future climate scenarios. Using >11,000 occurrence records and mid-century and late-century climate models (2050 and 2080 under moderate [SSP2-4.5] and high [SSP5-8.5] emissions), they found that climate change could profoundly shift vector ranges by 2080. Notably, while projections for 2050 showed relatively stable distributions, by 2080, a substantial expansion of suitable habitat is predicted in currently marginal areas, especially the Brazilian Amazonia and the deforestation arc of Amazonia. This suggests that warming temperatures and altered rainfall will enable certain vectors to colonize new regions (including rainforest fringe and previously cooler zones). Such expansion threatens to bring *T. cruzi* transmission to previously unaffected, vulnerable populations in these areas, highlighting the urgency of proactive surveillance and climate-adaptive control strategies in light of these forecasts.

In addition to continent-scale projections, finer-scale mapping has identified current hotspots of transmission risk using novel data sources. For example, Hill et al. (2024) harnessed citizen science (iNaturalist) records and MaxEnt modeling to map habitat suitability for three U.S. Southwest triatomine species (*T. protracta*, *T. rubida*, *T. recurva*) [125]. Over 700 geotagged bug observations were fed into ecological niche models with bioclimatic variables, and models pinpointed several potential transmission zones in the U.S. and northern Mexico, including coastal Southern California, the Sierra Nevada foothills, southern Arizona, and border regions of Texas/N. Mexico as having the highest human-triatomine encounter rates [125]. Interestingly, precipitation variables emerged as the strongest predictors of vector presence (e.g., rainfall in the warmest quarter for *T. protracta*, and in the driest quarter for *T. rubida* and *T. recurva*). Such analyses highlight how remote-sensing-derived climate data (temperature, precipitation indices) and crowdsourced observations can be combined to identify emerging risk areas far from traditional endemic zones [125]. The outputs inform targeted surveillance, e.g., public health agencies in Arizona and California can intensify monitoring and community education in the specific counties flagged by these suitability maps.

Moreover, researchers are refining mapping methods to improve prediction accuracy and account for data biases. Spatial modeling and GIS techniques have been used to delineate eco-regions and detect sampling gaps. Ceccarelli et al. (2020) employed clustering and regression-tree analyses on triatomine occurrence data to define biogeographic regions for New World triatomines, finding that environmental variables like precipitation, elevation, and vegetation cover best explain the partitioning of species distributions [126]. Such variables, obtained via remote sensing (e.g., satellite-derived vegetation indices, digital elevation models), are crucial inputs to distribution models and help capture the ecological niche of each species. This approach, combined with high-resolution climate surfaces, allowed researchers to compute the probability of future presence and identify regions where current expert-drawn range maps are likely to fail [127]. Nevertheless, Shirey and Rabinovich (2024) [128] found that many static range maps drawn from historical data will degrade in accuracy under climate change, as species move beyond their known ranges. Their analysis revealed significant gaps in sampling, as many areas where triatomines may already exist or invade remain under-surveyed, and calls for intensified field sampling in those zones to update distribution models. In summary, modern predictive mapping marries machine learning, niche modeling, and remote sensing to anticipate vector spread. These studies collectively demonstrate that climate change is poised to alter triatomine habitats, reinforcing the need for dynamic surveillance and predictive “risk maps” that can guide preemptive control efforts in both endemic and previously unaffected regions. A summary of studies using predictive mapping approaches is presented in Table 3.

### 4.2. Digital Surveillance Innovations

Geospatial analysis has been used to identify high-risk areas and improve resource allocation for CD. For example, a 2022 study in Texas mapped CD-related hospital diagnoses to find clusters of potential cases. Using GIS and 3D mapping of ICD-9/10 coded cardiomyopathy diagnoses, researchers pinpointed urban clusters where CD was likely underdiagnosed, highlighting the need for targeted surveillance and provider education in those hotspots [129]. Overall, GIS mapping combined with remote-sensing data has improved understanding of CD risk distribution and informed intervention planning in both endemic Latin America and emerging areas like the southern United States.

Moreover, researchers are increasingly applying machine learning (ML) to CD surveillance and risk prediction. In Brazil, a 2024 study developed an ML-based screening tool to predict individual infection risk using simple survey questions [130]. Among five algorithms tested, an AdaBoost model achieved an AUC ~0.77, and by adjusting the decision threshold, it could reach 100% sensitivity, potentially detecting all cases while saving ~22% in testing costs [130]. This demonstrates how ML can improve case finding and cost-effectiveness in human CD screening. Another effort used ML for spatiotemporal modeling of transmission. Ledien et al. (2022) compared boosted regression trees and random forests to a classical model for predicting the force-of-infection (FoI) across Colombia, suggesting ML can capture spatial trends (identifying areas of ongoing transmission) while highlighting where data gaps remain [131]. Machine learning has also been applied to vector identification, as one 2022 study trained a deep convolutional neural network on triatomine photos to distinguish kissing bugs from look-alike insects [132]. The model exceeded 94% accuracy on standard images and 91% even on crowd-sourced smartphone photos, proving robust to poor lighting and background noise [132]. This innovation could be integrated into surveillance apps so field workers or citizens can rapidly identify vectors from phone images, greatly expanding vector monitoring capacity.

Digital platforms have been developed to streamline CD surveillance and data management. The SisVetor system (piloted in 2022–2023) exemplifies an integrated vector surveillance software, facilitating real-time field data collection with geolocation of vector sightings, and it organizes the full surveillance workflow, from planning and execution to monitoring and control measures [133]. Early use in Brazilian municipalities indicates that such platforms can improve coordination between field teams and labs, replacing paper-based logs with centralized digital data. At the global level, WHO launched an open-source CD information system to aggregate multi-source data and provide interactive dashboards, compiling official case reports, surveillance records, drug distribution data, and more, in order to detect “epidemiological silence” (gaps in reporting) and display up-to-date disease metrics [134]. By visualizing case distributions and transmission routes in real time, such dashboards support better outbreak detection and help verify progress towards elimination.

One of the most significant trends since 2020 is the rise of citizen science projects that engage the public in CD vector surveillance. In Venezuela, researchers implemented the #TraeTuChipo (“Bring your kissing bug”) campaign in 2020 to compensate for a lapsed national program. Community members across the country submitted triatomine sightings via online surveys, social media, and text messaging, and in the first year, 79 triatomine specimens (predominately *P. geniculatus*) were reported from 18 states, with lab analysis confirming many carried *T. cruzi* (TcI strain) with blood meals from humans, chickens, and bats. This real-time digital surveillance by citizens effectively mapped the current vector distribution and infection risk despite an official data gap. In Brazil, a novel WhatsApp-based platform nicknamed “WhatsBarb” has similarly empowered the public. Through four annual campaigns up to 2024, volunteers sent photos/videos of suspected triatomines via the WhatsApp application [135]. Over approximately 5 years, the project amassed 465 insect records from 20 of 26 Brazilian states, although only ~32% were actual triatomines. Still, WhatsBarb greatly expanded the geographic scope of monitoring and catalogued over 100 insect taxa, providing feedback to participants and funneling confirmed bugs to health authorities [135]. The organizers conclude that this citizen surveillance approach can significantly aid detection and control of CD vectors, and could be replicated for other NTDs, potentially helping improve epidemiological insights (e.g., identifying emerging risk zones) that guide public health response.

### 4.3. One Health Integration

CD is increasingly understood through a One Health lens, which highlights the interconnected health of humans, animals, and their shared environments. This perspective recognizes that *T. cruzi* circulates among a wide range of hosts beyond humans, creating complex transmission cycles that require integrated control efforts. Wildlife reservoirs, including opossums, armadillos, rodents, and other mammals, play a pivotal role in maintaining these enzootic cycles, acting as ecological bridges between sylvatic and domestic environments [94]. Research in Mexico has shown that domestic dogs and wild opossums act as key reservoirs of *T. cruzi*, emphasizing the urgent need for coordinated surveillance and control strategies across veterinary and public health sectors [136]. A comprehensive review of American trypanosomiasis in southern Mexico further identified gaps in ecological data, wildlife monitoring, and electronic reporting, calling for cross-sectoral approaches that combine molecular diagnostics, animal surveillance, and environmental risk mapping [136]. From a One Health standpoint, *T. cruzi* infection also represents a direct threat to wildlife populations, as infection can lead to significant cardiac and systemic pathology in wild mammals, including marsupials, rodents, and nonhuman primates [137]. Infected wildlife not only sustains sylvatic cycles but may also experience morbidity that affects ecosystem balance and biodiversity. Thus, monitoring the impact of *T. cruzi* on wildlife health is a crucial yet underexplored aspect of One Health surveillance.

One Health relevance extends far beyond Mexico, as similar dynamics are reported across Latin America and even in non-endemic areas like the southern United States. In these regions, diverse wild and domestic animals, including opossums, raccoons, rodents, and dogs, harbor *T. cruzi* [138], sustaining sylvatic and peridomestic transmission cycles that make eradication impossible [94]. Surveillance work by Rodriguez et al. (2021) in El Paso County, Texas, and New Mexico detected high infection prevalence in triatomine vectors (66.7%), feral dogs (45.3%), cats (29.2%), and wild animals (71.4%), with bloodmeal analyses confirming feeding on humans, dogs, and wildlife, providing strong evidence of active enzootic and potential zoonotic transmission [9]. These findings reinforce the ecological interdependence of humans, vectors, and wildlife and highlight how environmental disturbances, such as deforestation and habitat fragmentation, can increase contact between infected reservoirs and human populations [21]. Recent field studies in Texas revealed high *T. cruzi* prevalence in *Triatoma sanguisuga* bugs collected from dog kennels, with bloodmeal analysis confirming feeding on dogs, wildlife, and humans, and parasite genotyping detecting both TcI and TcIV strains [92]. Dogs have also been highlighted as valuable sentinel species, with recent surveys documenting infections in both northern and southern Mexico, linking canine health directly to human transmission risk [139].

Environmental factors further complicate the picture, as land-use changes and biodiversity loss have been linked to higher infection rates among wildlife reservoirs, increasing the likelihood of spillover to vectors and humans [140]. Addressing these eco-epidemiological connections through wildlife surveillance and environmental management is therefore essential to any One Health strategy for CD. Major health organizations, including the World Health Organization (WHO), now advocate for embedding One Health principles into CD control programs to address these intertwined drivers of disease spread. Embracing this framework and uniting human medicine, veterinary science, entomology, and ecology is essential to disrupt transmission networks, protect vulnerable populations, and achieve sustainable, long-term control of CD on a global scale. Recent studies applying a One Health framework to CD are summarized in Table 4.

## 5. Recommendations

Building upon the evidence presented across the main sections of this review, we propose the following recommendations to strengthen research, surveillance, and control strategies for Chagas disease (CD) under a One Health framework:

Global Spread—Strengthen Cross-Border Surveillance and Health System Integration

Countries of endemic to non-endemic regions should prioritize the implementation of universal screening protocols for blood donors and pregnant women, along with expanded diagnostic access for underserved populations. Bilateral and regional agreements, especially between Latin America, North America, and Europe, are essential to harmonize data reporting, case management, and surveillance practices. Integrating Chagas disease into existing primary healthcare systems will be key to identifying hidden reservoirs of infection, improving long-term disease control outcomes.

Ecological Shifts—Integrate Environmental and Wildlife Surveillance

Vector-control programs must adapt to the ongoing ecological transitions driven by deforestation, urban expansion, and climate change. We recommend expanding entomological and ecological monitoring networks to include sylvatic and peridomestic habitats, with special attention to emerging vector species such as *T. rubida* and *T. sanguisuga*. Wildlife health surveillance should be integrated into national strategies, recognizing *T. cruzi* as a pathogen of both veterinary and ecological relevance. Land management and biodiversity conservation policies should be coordinated with public health objectives to reduce spillover risk.

Research Frontiers—Foster Innovation in Diagnostics and Digital Epidemiology

Continued investment is needed in field-deployable molecular tools, such as LAMP and CRISPR-based assays, to improve early detection and monitoring in low-resource settings. In parallel, digital epidemiology platforms such as serofoi and other data-driven modeling tools should be leveraged to predict hotspots and track infection dynamics. Funding agencies and governments should support open-access databases that integrate human, animal, and vector data to enhance real-time decision-making.

One Health Implementation—Promote Intersectoral Collaboration

To achieve sustainable control, ministries of health, agriculture, and environment must adopt coordinated frameworks for surveillance, vector control, and food safety. International organizations (e.g., WHO, PAHO, OIE) should support capacity-building programs to train multidisciplinary teams in One Health-based CD management.

Collectively, these recommendations emphasize that the sustainable control of CD depends on bridging epidemiological surveillance, ecological understanding, and technological innovation within an integrated global health strategy.

## 6. Conclusions

Chagas disease has transitioned from a historically rural, Latin American parasitic illness to a complex global health challenge. This review highlights how human migration, ecological disruption, and weakened control programs have contributed to the emergence of new geographic hotspots and increased impact of oral and congenital transmission routes. Climate change and land-use changes are further expanding the ecological range of triatomine vectors, creating new risks even in non-endemic regions. These dynamic shifts underscore the urgent need for sustained surveillance, political commitment, and public health investment to prevent the resurgence and global spread of the disease.

Recent scientific advances between 2020 and 2025, including vector genomics, climate-based predictive modeling, innovative diagnostic platforms, and digital surveillance tools, provide powerful opportunities to improve disease detection and control. Integrating these tools within a One Health framework, which links human, animal, and environmental health, will be critical for effective intervention strategies. Moving forward, global cooperation and interdisciplinary collaboration will be essential to break transmission cycles, enhance early detection, and ultimately reduce the burden of CD on vulnerable populations worldwide.

## Figures and Tables

**Table 1:** Summary of major transmission routes of *T. cruzi* (CD).

Route	Mechanism	Predominant Regions	Key Data	Public Health Impact
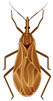 Vectorial	Feces of infected triatomines contaminate skin/mucosa	Rural Latin America (Bolivia, Gran Chaco, NE Brazil)	Prevalence > 6% in Bolivia; control success in Southern Cone, but insecticide resistance rising	Still a driver of chronic burden; risk of resurgence
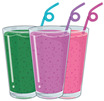 Oral	Contaminated food/beverages (açaí, sugarcane, guava)	Amazonia (Brazil, Venezuela, Colombia, French Guiana)	>70% of acute cases in Amazonia; outbreaks up to 100+ cases; CFR 10–35%	Aggressive acute disease (fulminant myocarditis, systemic inflammation)
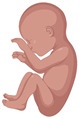 Congenital	Mother-to-child (transplacental)	Bolivia, Brazil, Paraguay, Colombia; migrants in Spain, Italy, U.S.	8000–15,000 cases/year; 1–10% transmission; 5–7% in Bolivian/Brazilian migrants	Nearly 100% curable if detected early; WHO 2030 elimination target
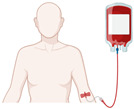 Transfusional	Infected blood products	Latin America (historic); risk in U.S./Europe without screening	Screening widespread in endemic countries; patchy in others	Sporadic risk where screening inconsistent
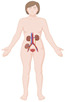 Transplantation	Infected organ donors	U.S., Spain, Italy, Switzerland, Japan	Isolated cases; often underrecognized	High mortality in immunosuppressed if undetected
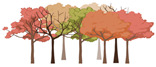 Sylvatic/Spillover	Contact with infected vectors or reservoirs	Southern U.S. (Texas, New Mexico), Amazonia Basin, Gran Chaco, and other endemic rural regions of Latin America.	Infected vectors (*Triatoma* spp.) and dogs/cats documented; human cases rare	Emerging zoonotic concern, climate change may expand risk

**Table 2:** Summary of Omics Studies on Triatomine Vectors (2020–2025).

Reference	Type	Vector Species	Approach	Key Findings
Traverso et al., 2022 [117]	Transcriptomics—Insecticide Response	*Triatoma infestans*	RNA-seq after sub-lethal deltamethrin exposure	Differential expressions of ABC transporters, heat shock proteins, odorant-binding proteins, and cuticle-related genes. Highlights pathways linked to detoxification and insecticide resistance.
Praça et al., 2022 [118]	Sialomics/Proteomics—Salivary Glands	*Triatoma sordida*	Integrated proteomics & transcriptomics	>26,000 salivary transcripts and 132 secreted proteins identified. Lipocalins were dominant (~89% of secretory output), along with apyrases and protease inhibitors, showing mechanisms of anticoagulation and immune evasion.
Peterson et al., 2024 [115]	Genomics	*Triatoma sanguisuga*	Whole-genome sequencing	First high-quality genome assembly (1.16 Gb, ~17,799 predicted genes, 99.1% BUSCO completeness, 61% repetitive DNA). Foundation for studies on blood-feeding, host-seeking, and vector competence.
Barbosa et al., 2024 [120]	Comparative Proteomics—Immune Factors	*Rhodnius prolixus* vs. *R. colombiensis*	Comparative hemolymph & saliva proteomics	*R. prolixus* had higher levels of trypanolytic molecules (lysozyme, prolixin, nitrophorins, serpins). Correlated with ability to lyse TcII strains (Y strain). Highlights vector-parasite immune interactions and evasion mechanisms by TcI strains.
Duan et al., 2025 [116]	Transcriptomics—Developmental	*Triatoma rubrofasciata*	RNA-seq across developmental stages	Identified stage-specific gene expression. Venom-like salivary proteins (e.g., histidine phosphatase, serine carboxypeptidase) upregulated in late nymphs. Adult stage showed high CYP425A1 expression, indicating detoxification adaptation.

**Table 3:** Summary of Studies Using Climate-Based Predictive Mapping to Assess Triatomine Vector Distribution (2020–2025).

Reference	Geographic Scope	Approach	Key Findings
Ceccarelli et al., 2020 [126]	New World triatomines (multiple countries)	Clustering and regression-tree analyses of occurrence data with remote-sensing variables (elevation, vegetation indices, precipitation)	Defined biogeographic regions and identified environmental factors influencing species distributions, providing an environmental baseline for niche modeling.
Hill et al., 2024 [125]	Southwestern U.S. & Northern Mexico (*T. protracta*, *T. rubida*, *T. recurva*)	Citizen science data (iNaturalist) integrated with MaxEnt modeling; >700 geotagged observations with remote-sensing climate variables	Identified high-risk zones in Southern California, Sierra Nevada foothills, Southern Arizona, and Texas/N. Mexico border. Precipitation was the strongest predictor of vector presence.
Shirey & Rabinovich, 2024 [128]	Global focus, with emphasis on Latin America	High-resolution climate surfaces combined with Bayesian additive regression trees (BART)	Demonstrated that static expert-drawn maps degrade under climate change as species move beyond known ranges. Highlighted sampling gaps, stressing need for intensified field surveys.
Brasil et al., 2025 [69]	Latin America (55 triatomine species)	Ensemble ecological niche modeling using machine learning algorithms (MaxEnt, Random Forest); >11,000 occurrence records; climate scenarios SSP2-4.5 & SSP5-8.5 for 2050 and 2080	By 2050, distributions remained mostly stable. By 2080, major expansion of suitable habitats, especially in the Brazilian Amazonia and deforestation arc, increasing risk to previously unaffected populations.

**Table 4:** Summary of Studies on One Health integration Approaches for CD (2020–2025).

Study	Geographic Scope	Components Integrated	Key Findings
Rodriguez et al., 2021 [9]	El Paso County, Texas & New Mexico, USA	Triatomine vectors/Feral dogs and cats/Wild animals	Found high *T. cruzi* prevalence: 66.7% of triatomines, 45.3% of feral dogs, 29.2% of feral cats, and 71.4% of wild animals were infected. *Triatoma rubida* was the dominant species (98.2%). Bloodmeal analysis identified humans, dogs, cats, and wildlife as feeding sources, suggesting active enzootic and potential zoonotic transmission.
Velázquez-Ramírez, Pérez de León & Ochoa-Díaz-López, 2022 [136]	Chiapas & Oaxaca, Mexico	Domestic animals/Wildlife/Humans/Ecology	Wildlife maintain *T. cruzi* in nature; domestic animals also act as reservoirs; environmental & ecological gaps in surveillance identified; need for molecular diagnostics and electronic data reporting.
Busselman & Hamer et al., 2022 [94]	Southern USA	Wildlife reservoirs (opossums, rodents, raccoons, etc.), domestic dogs, triatomines, human dwellings	Quantitative synthesis shows many animal species are infected; triatomines’ bloodmeal analysis implicates human/domestic hosts; highlighting underrecognized transmission risk and diagnostic gaps.
Dávila et al., 2024 [139]	Northern & Southern Mexico	Dogs (domestic animals)/Humans (shared spaces)/Vector interface	Dogs found infected in various regions; dogs share habitats with humans; suggests utility of using dogs as sentinel/reservoirs to monitor transmission risk.
Velázquez-Ramírez et al., 2025 [141]	Mexico	Human/Domestic animals/Vectors/Environment	Surveys and data show domestic animals and wildlife carry *T. cruzi*; vector control and surveillance strategies need to integrate all sectors. Emphasis on surveillance in rural and semi-rural regions.

## Data Availability

Data sharing does not apply to this article, as no datasets were generated or analyzed during the current study. Figures were created in BioRender.com (accessed on 25 August 2025).

## Abbreviations

The following abbreviations are used in this manuscript:

CDC	Centers for Disease Control and Prevention
CFR	Case Fatality Rate
DNA	Deoxyribonucleic Acid
DTU	Discrete Typing Unit
ELISA	Enzyme-Linked Immunosorbent Assay
EU	European Union
GIS	Geographic Information System
IFA	Immunofluorescence Assay
LC-MS	Liquid Chromatography–Mass Spectrometry
LAMP	Loop-Mediated Isothermal Amplification
ML	Machine Learning
NGS	Next-Generation Sequencing
PCR	Polymerase Chain Reaction
RDT	Rapid Diagnostic Test
RNA	Ribonucleic Acid
SSP	Shared Socioeconomic Pathway
WHO	World Health Organization
